# Promoting Daily Well-being in Adolescents using mHealth

**DOI:** 10.1007/s10964-022-01656-8

**Published:** 2022-07-22

**Authors:** Michelle M. J. Mens, Loes Keijsers, Evelien Dietvorst, Soldado Koval, Jeroen S. Legerstee, Manon H. J. Hillegers

**Affiliations:** 1 Department of Child and Adolescent Psychiatry/Psychology, Erasmus MC University Medical Center Rotterdam, Rotterdam, The Netherlands; 2 Department of Epidemiology, Harvard T.H. Chan School of Public Health, Boston, MA USA; 3 Department of Psychology, Education & Child Studies/Clinical Child and Family Studies, Erasmus University Rotterdam, Rotterdam, The Netherlands

**Keywords:** Coping, COVID-19, Ecological momentary assessment, EMA, mHealth, Well-being

## Abstract

Adolescents are at increased risk for developing mental health problems. The Grow It! app is an mHealth intervention aimed at preventing mental health problems through improving coping by cognitive behavioral therapy (CBT)-inspired challenges as well as self-monitoring of emotions through Experience Sampling Methods (ESM). Yet, little is known about daily changes in well-being and coping during a stressful period, like the COVID-19 pandemic. The current study aimed to elucidate daily changes in positive and negative affect, and adaptive coping, and to better understand the within-person’s mechanisms of the Grow It! app. The sample consisted of 12–25-year old Dutch adolescents in two independent cohorts (cohort 1: *N* = 476, Mage = 16.24, 76.1% female, 88.7% Dutch; cohort 2: *N* = 814, Mage = 18.45, 82.8% female, 97.2% Dutch). ESM were used to measure daily positive and negative affect and coping (cohort 1: 42 days, 210 assessments per person; cohort 2: 21 days, 105 assessments). The results showed that, on average, adolescents decreased in daily positive affect and adaptive coping, and increased in their experienced negative affect. A positive relation between adaptive coping and positive affect was found, although independent of the CBT-based challenges. Latent class analysis identified two heterogeneous trajectories for both positive and negative affect, indicating that the majority of participants with low to moderate-risk on developing mental health problems were likely to benefit from the Grow It! app.

## Introduction

Adolescence refers to the transition from childhood to adulthood, which is typically from the onset of puberty to guardian independence (12–25 years) (Jaworska & MacQueen, 2015). This period is a unique opportunity for personal growth and development as it coincides with social changes, spending less time with parents and more time with peers, resulting in an increase in autonomy, independence, identity, and self-awareness (Ciranka & van den Bos, 2019). At the same time, about one in five adolescents experiences emotional problems, including depressive symptoms, highlighting the vulnerability of this age group relative to other age groups (Kessler et al., 2005). Like any other human being, adolescents have fundamental needs for safety (Crandall et al., 2020), feeling belonged (Crandall et al., 2020), and a sense of purpose (Poole & Evans, 1989). When these needs are violated, one can experience the flipside of these needs, including feeling threatened, isolated, and useless, which leads to an increase risk on developing depressive symptoms (Crandall et al., 2020). The setting in which adolescents meet these fundamental needs rely, among other things, on parental support (Janssen et al., 2021), close friendships (Berndt, 2002), and involvement in school and communities (Clemens et al., 2020). Extreme external factors, such as the imposed government restrictions on COVID-19, have made it challenging for youth to socialize with peers to develop their independence, as well as cope with difficult situations where social interactions are naturally supportive (Keijsers & Bülow, 2021). For example, whereas previously youth with the strongest cards made it through adolescence without too many problems, this group may also run an increased risk of developing emotional problems as about 30–50% of adolescents were feeling lonely as of social deprivation (Loades et al., 2020). Moreover, certain subpopulations, including those from minority racial-ethic background (Smith et al., 2020), females (Ma et al., 2021) and individuals with pre-existing mental health problems (Gobbi et al., 2020), experience more impact on their mental health due to COVID-19. In addition, imposed social restrictions may have been harder hit by older adolescents, as they are more likely to participate in social events (such as parties, gatherings) and form intimate relationships that provide safety, comfort and feeling belonged. Indeed, previous epidemiological studies indicate that older adolescents experience more mental health issues due to COVID-19, such as anxiety, compared to younger adolescents (Duan et al., 2020). Overall, the serious threat to the mental health of adolescents is shown by the worrying number of youth affected by mental health disorders, such as anxiety and depression, which amounts to nine million adolescents in Europe, according to UNICEF (UNICEF, 2021). It is clear that the demand for clinical care far exceeds the supply (American Psychological Association, 2020). Delays in treatment may have deleterious consequences regarding long-term mental health problems (de Girolamo et al., 2012). Prevention and early detection of mental health problems are necessary to reduce the burden, hence less invasive treatments aimed at preventing adolescents from decreasing their emotional well-being receive attention. In particular, mobile health (mHealth) applications can offer a unique opportunity to provide mental health support in a very accessible way since there are no geographically, financially or socially barriers (Price et al., 2014). Therefore, this study aimed to examine an mHealth intervention on promoting daily well-being in adolescents during a stressful period.

### mHealth and CBT

mHealth apps are technology-based solutions to support mental health (Andersson & Titov, 2014). In addition to contributing to social support during times of social constraints, like COVID-19, such technology can integrate components aimed at increasing adaptive coping and reducing symptoms of mental illness (Chandrashekar, 2018). Adaptive coping, defined as a problem-solving adaptation when faced with stressful situations, can provide a crucial buffer against the negative impact of the pandemic on daily well-being (Flesia et al., 2020). By periodically reporting thoughts, behaviors, and actions, mHealth app users can increase their emotional self-awareness, which has been found to be involved in mental health (anxiety, depressive symptoms), and improve coping (Bakker et al., 2016). In addition, mHealth appears to be most successful when it provides stress management interventions (Bakker et al., 2016).

In this context, there is a growing interest in using the principles of cognitive-behavioral therapy (CBT) in an mHealth setting (Bakker et al., 2016). CBT is an effective technique for preventing or treating mental illness by controlling maladaptive thoughts that arise because of negative interpretation situations (Luo & McAloon, 2021). Key principles of CBT include; context engagement (promote adaptive imagining and execution of new experiences), attention change (to change the focus of attention on relevant, non-disturbing stimuli, such as acceptance.), and cognitive change (to change the perspective of an event which changes the emotional significance and meaning of that event) (Mennin et al., 2013). Randomized control trials estimating the efficacy of CBT-based interventions through mHealth have been shown to reduce depressive symptoms and social anxiety disorders (Bakker et al., 2016).

### mHealth and Experience Sampling Methods

While prospective cohort studies examining the psychological impact during the COVID-19 pandemic show an increase in anxiety and depressive symptoms and a decrease in overall life satisfaction, especially in adolescents (Magson et al., 2021), little is known about the daily emotional well-being of adolescents. In our mHealth application, we have embedded the experience sampling method (ESM) to monitor and study the everyday changes in well-being and coping. To sample the real-time experiences of adolescents, players report how they feel, behave, act, etc., at multiple random times a day by completing micro-questionnaires (Stone & Shiffman, 1994). The resulting intensive longitudinal data, a series of “emotional snapshots”, provide detailed scientific insights about individual’s experiences throughout the day.

This repeated real-time measurement has several advantages, both from the perspective of the user, as from the perspective of the researcher. At first, ESM has a higher ecological validity as it is less prone to recall bias compared to traditional self-reports (van Roekel et al., 2019). Secondly, ESM can be used to obtain time series data which allows an *N* = 1 test of the effectiveness of an intervention. Where historically, the randomized control trial (RCT) is considered the gold standard for testing interventions, creating a group-based comparison that controls for differences between individuals by random assignment to a treatment, *N* = 1 designs have gained popularity (Blackston et al., 2019). In such a within-person design, individuals are compared before, during, and after the treatment, and thus serves as their own “control condition”. When such an *N* = 1 approach is conducted among a large sample, pre-existing differences between individuals can be controlled for in multilevel statistical analyses. As such, changes at the mean-level in participants’ outcomes from before to after intervention are tested, which cannot be explained by how individuals differ before the intervention. Moreover, this allows to test how individuals differ(i.e., effect heterogeneity: (Bolger et al., 2019) and to express for how many individuals the interventions work (Grice et al., 2020). Thirdly, ESM has demonstrated potential for clinical applications, such as improving self-awareness and self-monitoring (Folkersma et al., 2021). Gaining individualized insight into mood and behavior patterns can lead to a more streamlined (self-)management of emotional problems (van Os et al., 2017). This self-management, here aided by gamified online apps, has been suggested as one way to reduce an increased burden on clinical care (Bos et al., 2019), and is therefore potentially interesting during COVID-19.

### The Grow It! app

With the prevalence of depression and anxiety increasing, and health services being no longer able to meet the demands, there is an urgent need for preventive tools. Grow it! is an mHealth app developed and tested for adolescents (12–25) to prevent mental health problems using two key components (Dietvorst, Aukes, et al., 2022). First, using ESM participants report their emotions five random times a day, which can improve reflection and self-insights. Second, the Grow It! app offers daily CBT-inspired challenges, aimed at promoting adaptive coping by focusing on aspects such as social support, self-acceptance, positive distraction, and problem solving (Dietvorst, Aukes, et al., 2022). Prior research has indicated good user evaluations (score 7.1–7.2 of 10). In an initial study among users, 20.6–44.2% reported that the CBT-based challenges made them more active and 66.8–72.4% reported to become more reflective of their own emotional well-being as a result of the ESM (Dietvorst, Aukes, et al., 2022). Moreover, over the course of three to six weeks, mean level of affective and cognitive well-being increased, and depressive symptoms decreased (Dietvorst et al. 2022). Even though this first proof of concept suggests potential effectiveness, it is unknown whether adolescents’ daily emotional experience and adaptive coping improves while playing the app, and to which extent improvements in well-being can be explained by engaging in CBT-based challenges.

## Current Study

The current preregistered longitudinal study had two aims. The first aim was to estimate the over-time within-person changes in daily well-being while playing the Grow It! app during three to six weeks of the first two waves of the COVID-19 pandemic. The second aim was to better understand the within-person’s mechanisms of CBT-based challenges on improving coping and subsequent well-being. To examine every day well-being, we used positive and negative affect, which are self-report measures that assess independent constructs ranging from low to high levels of everyday emotional experience (Watson et al., 1988). Based on previous Grow It! studies (Dietvorst, Aukes, et al., 2022; Dietvorst et al. 2022), it was expected that the Grow It! app is effective, as reflected an increase in the mean level of positive affect and a decrease in the mean level of negative affect over the course of the study. This study further investigated without a priori hypotheses whether heterogeneous trajectories (i.e., subgroups of individuals with a similar rate of change) would exist who would differ on age, sex, depressive symptoms, anxiety, affective well-being, adaptive coping at baseline and receiving psychiatric care. In addition, as one of the hypothesized working mechanisms of the app, the daily offered CBT-based challenges were expected to promote adaptive coping in the Grow It! app users. Therefore, it was hypothesized that everyday mean level of adaptive coping would increase over the course of the study. Furthermore, a positive relation between within-person changes in adaptive coping and within-person changes in positive affect was expected. Finally, the within-person association between playing CBT-based challenges and positive affect was expected to be mediated (i.e., partially explained) by adaptive coping.

## Method

### The intervention: Grow It! app

The Grow It! app is a multiplayer serious gaming smartphone tool available on both Android and iOS (Dietvorst, Aukes, et al., 2022). The app is developed for 12–25 year old Dutch adolescents at low to moderate risk for developing mental health symptoms. Adolescents fill out ESM questionnaires five random times per day, and complete daily CBT-based challenges which should activate more active coping skills (e.g., social support, acceptance, problem solving, and distraction). For example, adolescents are challenged to take a picture of two red cars parked together (a way to activate children to go outside) or to give a compliment to a friend (to activate social support). To personalize the experience, each day, users can choose one of three unique challenges. The gamify the experience, the apps contains key-elements from game design. Motivation is enhanced by competition, and collaborating in teams, against other teams. To this end, participants are randomly assigned to a team of four to six players. The goal of the team is to grow a virtual tree with embellishments, for which they must use the earned points. Points can be earned both by filling out ESM questionnaires and by completing challenges. Team members can communicate and support each other by posting positive stickers in the chat function. The app’s privacy and security were approved by the privacy and security office of Erasmus MC, and the app complies with the Dutch General Data Protection Regulation (GDPR) and NEN-norm 7510:2017 (Dutch standard of information security management systems in healthcare).

### Sample and Procedure

**Fig. 1:**
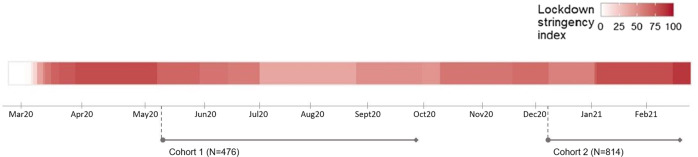
Illustration of confirmed COVID-19 cases and stringency of governmental measures in the Netherlands. Enrollment of participants of cohort 1 was between May 11th 2020 and June 9th 2020. During the first cohort, the Dutch government announced a series of measures that had an enormous impact on the social capacity of citizens, including schools that have started teaching remotely, closing sports clubs, encouraging people to stay at home and minimalizing the number of visitors. From June 2020, measures were slightly eased by re-opening of primary and secondary schools, sport clubs and restaurants with a limited number of visitors. Enrollment of cohort 2 was between December 14th 2020 and January 25th 2021. During the second cohort, a lockdown has been confirmed with strict measures including distance learning, closure of non-essential shops and curfews

This study was conducted in two independent cohorts, in cohort 1 (first lockdown) users played the Grow It! app for six weeks, and in cohort 2 (second lockdown), users played for three weeks (Fig. 1). In cohort 1, 1220 participants completed the baseline questionnaires, and in cohort 2, 1994 participants did so. In cohort 1, out of 1220 that completed the baseline questionnaire, 353 did not activate the Grow It! app on their smartphone and were excluded. In cohort 2, out of 1994 that completed the baseline questionnaire, 428 did not activate the Grow It! app on their smartphone and were excluded. In the current study, we maintained the following exclusion criteria of an ESM compliance of less than 5% (cohort 1: <10 ESM/max 210 ESM, cohort 2: <5 ESM/max 105 ESM); and playing fewer than two CBT-based challenges over the course of the study (Table S1 and Table S2). The first cohort was composed of 476 participants (23,928 ESM observations; average of 50.3 per person (out of max. 210)) and the second of 814 participants (29,607 ESM observations; average of 36.3 per person (out of max. 105)). In cohort 1, the mean age at baseline was 16.24 years (SD (±) 3.01), of which 362 (76.1%) were girls, and 422 (88.7%) Dutch. In cohort 2, the mean age at baseline was 18.45 years (± 3.44), of which 671 (82.%) were girls, and 791 (97.2%) Dutch.

During the first lockdown (May 2020-Aug 2020), schools started teaching remotely, sports clubs were closed, and people were encouraged to stay at home and minimize the number of visitors. During the second lockdown (December 2020-March 2021), strict measures were maintained, including online learning, closure of non-essential shops, sport clubs and curfews. Over the course of this first year of the pandemic, studies have now established a decrease in functioning (Loades et al., 2020), more loneliness, more family stress and burden (Weeland et al., 2021). A recent study which followed youth for a full year during the pandemic with intensive measures suggests the second lockdown worsened the situation for adolescents (Buist et al., 2022).

### Procedure and Ethics

Participants were recruited via advertisements on (social) media and through online announcements by schoolteachers. Inclusion criteria were owning a smartphone, between 12 and 25 years, and being able to understand Dutch. First, participants registered via the website (www.growitapp.nl/corona) where they were asked to sign an online informed consent form. For participants aged 12–16 years, their parents were also asked to sign an online informed consent form. The Grow It! app study has been approved by the Ethics Committee of the Erasmus Medical Center (registration number: MEC2020-0287). This longitudinal study used both baseline questionnaires and ESM data to estimate the changes in positive and negative affect, as well as in adaptive coping. After an online baseline questionnaire of one-two minutes, participants received a text message with a unique code to login on the Grow It! app on their smartphones. All measures can be found online. The current study includes a selection of items related to specific research questions.

#### Sampling scheme

The ESM micro-questionnaires were sent out to participants five times a day in a semi-random design and notified at random time points within a fixed time interval between 09.00 h and 21.00 h (45 min time window to respond). Reminders were sent after 40 min. Participants did receive ESM questionnaires during school hours, but they were able to mute their phones. The total 90 min time to complete the questionnaire also ensured that it did not interfere with the class and could be completed during school breaks. A total of 210 ESM questionnaires (5 ESM/day*42 days) were sent out in cohort 1, and 105 (5 ESM/day*21 days) in cohort 2. A detailed description of the ESM questionnaires can be found online. As a reward system, a random group of participants, with at least 70% compliance, was drawn monthly to win a gift voucher worth €75, €50, €30 or €15 or Apple Airpods. The mean ESM compliance rate, which is defined as the percentage of ESM questionnaires that has been filled out, was 23.9% (± 22.7) in cohort 1, and 34.6% (± 25.6) in cohort 2 (Table 1). The average completion time to fill out the ESM questionnaires was for cohort 1: 1 min and 42 sec (± 3 minute and 20 sec) and cohort 2: 2 min and 0 sec (± 2 min and 34 sec).

**Table 1:** Baseline characteristics

	Cohort I (*n* = 476)	Cohort II (*n* = 814)	*P* value of group difference
Age (years), mean (SD)	16.24 (3.01)	18.45 (3.44)	<0.0001
Female, *n*(%)	362 (76.1%)	671 (82.8%)	0.0055
Ethnicity			0.0002
Dutch	422 (88.7%)	791 (97.2%)	
Non-Dutch	2 (0.4%)	2 (0.24%)	
Mixed	35 (7.4%)	16 (2.0%)	
Education			
Primary school	9 (1.9%)	7 (0.9%)	
Secondary school			
*low*	30 (6.3%)	48 (5.9%)	
*middle*	70 (14.7%)	110 (13.5%)	
*high*	241 (50.6%)	173 (21.3%)	
College/University			
*low*	18 (3.8%)	89 (10.9%)	
*middle*	32 (6.7%)	146 (17.9%)	
*high*	35 (7.4%)	168 (20.6)	
Other	2 (0.4%)	50 (6.1%)	
COVID related			
Diagnosed positive or symptoms	42 (8.8%)	107 (13.1%)	0.0004
Family member affected	54 (11.3%)	153 (18.8%)	<0.0001
Depression (score), mean (SD)	5.41 (3.98)	7.56 (4.47)	<0.0001
Anxiety (score), mean (SD)	16.24 (4.67)	18.91 (4.43)	<0.0001
Wellbeing (score), mean (SD)	4.92 (1.28)	4.26 (1.40)	<0.0001
Adaptive coping (score), mean (SD)	4.23 (1.33)	4.04 (1.26)	0.013
Psychological care			0.059
*Yes*	65 (13.7%)	172 (21.1%)	
*Waiting list*	10 (2.1%)	40 (4.9%)	
ESM compliance (%), mean (SD)	23.94 (22.70)	34.62 (25.64)	<0.0001
Challenge compliance (%), mean (SD)	40.74 (26.87)	54.39 (28.39)	<0.0001
Average daily positive affect (score), mean (SD)	4.92 (1.33)	4.40 (1.41)	<0.0001
Average daily negative affect (score), mean (SD)	1.94 (1.04)	2.17 (1.18)	<0.0001

Variables are expressed as mean (SD), or percentage (%). Difference between two cohorts based on Student t test

### Measures

#### Baseline characteristics

##### Social Demographics

In the online baseline questionnaire, participants were asked about their sex (female/male/other), age (years), ethnicity (Dutch/Non-Dutch/Mixed), whether participants received psychological care or were on a waiting list, and their educational level, including primary school, low = (preparatory school for) technical and vocational training, middle = (preparatory school for) professional education, high = (preparatory school for) university).

##### Depressive symptoms

Adolescent depressive symptoms were assessed in the baseline online questionnaire. Items were based on the Children’s Depression Inventory (Kovacs, 1985). A total of 12 items were used. Each item has three statements from which the adolescent could choose the one that would best describe him/her over the last week. For example: ‘I am sad sometimes’ (0), ‘I am often sad’ (1), and ‘I am sad all the time’ (2). Total scores ranged from 0 to 24, with higher scores indicating more depressive symptoms.

##### Anxiety symptoms

In the baseline questionnaire, anxiety was assessed using nine items based on the Screen for Child Anxiety Related Disorders (SCARED) (Birmaher et al., 1997). Subjects were asked to choose one that would best describe their feelings over the last two weeks. For example: “In the last 2 weeks, I was worried whether other people would like me”. Answer categories ranged from “not at all” (0), “a little bit/sometimes” (1), “definitely/often” (2). High scores indicated more anxiety.

##### Affective well-being

Affective well-being was based on the item: “How happy did you feel in the past week?”. Answer categories were based on a seven-point Likert scale anchored “at not at all” (1) to “very” (7) (Office for National Statistics, 2018).

##### Mean adaptive coping

For baseline adaptive coping score, the first assessed mean score of the ESM adaptive coping items were used. For cohort 1, four items were used whereas for cohort 2, five items were used. Items were based on validated questionnaires (Cracco et al., 2015; Gullone & Taffe, 2012; Jermann et al., 2006). For example: “I thought the situation also had positive sides”, “I thought I had to accept it”. Answer categories were based on a seven-point Likert scale anchored at “not at all” (1) to “very”(7).

#### ESM assessment

##### Positive and negative affect

Affective well-being was assessed based on the Positive and Negative Affect Schedule (PANAS) (Watson et al., 1988). The ESM questionnaire asked participants about their daily affective wellbeing five times a day (seven-point Likert scale anchored at “not at all” (1) to “very” (7)) (Watson et al., 1988). To this end, daily positive affect was based on the mean of the following items: “Right now, I feel relaxed/ satisfied/happy/confident.” Daily negative affect was based on the mean of the following items: “Right now, I feel mad/stressed/irritated/sad”.

##### Daily adaptive coping

Participants were asked to think about a negative event they had experienced at the end of each day. In this context, coping strategies were measured based on the mean of four (cohort 1)/five (cohort 2) items that were derived from validated questionnaires (seven-point Likert scale anchored at “not at all” (1) to “very” (7)) (Cracco et al., 2015; Gullone & Taffe, 2012; Jermann et al., 2006). Daily coping was based on the mean of the following items: “I thought the situation also had positive sides”, “I thought I had to accept it”, “I told someone how I was doing”, “I did something nice”, “I thought about how to solve it”.

##### CBT-based challenges

Participants were invited to complete one out of three offered daily CBT-based challenges from the Grow It! app, and we registered whether or not they had done so that day.

##### Time-varying covariates

To adjust for external factors that could affect emotional well-being, daily COVID-19 stringency Index and daily weather were included as covariate. Daily governmental measures in the Netherlands were measured, using the COVID-19 stringency Index (0 to 100) (Hannah et al. 2020) and daily weather with daily maximum temperature as proxy (Dutch weather institute) (KNMI, 1993).

#### Statistical analyses

##### Preregistered analyses

The analysis plan was preregistered prior to data analyses. As multiple assessments of each individual were obtained, multilevel models, also known as linear mixed effects models (LMM) were conducted in R (Version 4.0.2). LMM analyses were done using the lme4 package (Bates et al., 2015). Latent growth models were conducted using the lcmm package (Proust-Lima et al., 2017). In all models, time constant predictors were grand-mean centered, including age and sex. Time-varying predictors were person-mean centered, including time, daily COVID-stringency index, and daily weather. For centering the package misty was used (Yanagida, 2022).

To address the first study aim and to identify the within-person change over time in positive- and negative affect, adaptive coping during two separate lockdowns during the COVID-19 pandemic, three separate linear mixed models (LMM) were generated as preregistered. The main predictor was time (recoded to a week scale). Models were controlled for age, sex, COVID stringency index, and weather. Because the duration of the intervention and the period in which the study started differed between the two cohorts, the analysis were independently performed in each cohort.

A stepwise approach per outcome (positive affect, negative affect, coping) was used. First, an intercept-only model was conducted to assess the relative amount of between- and within-person variance in the outcome. Second, an unconditional growth model with time as predictor was specified (random intercept and random slope). Third, the fixed effects of time-varying covariates and interaction terms between the time-varying covariates and time were added.

Furthermore, as an exploratory preregistered analysis, to identify potential different subgroups on trajectories of positive and negative affect within the population, latent class growth models were used. This technique allows to classify individuals according to their most likely trajectory. Both 1 and 2 classes were applied (*n* = 50 iterations). To compare which model would best describe the underlying patterns in our data, multiple statistical fit indices were used, including Akaike Information Criteria (AIC), Bayesian Information Criteria (BIC), sample size adjusted Bayesian Information Criteria (ssaBIC). To assess classification accuracy, posterior probability, and entropy values were inspected, a full explanation of this technique see (van de Schoot, 2015). The posterior probabilities represent the mean of a subject assigned to a particular class, values closer to 1.0 are desirable. Entropy indicates how accurately the model defines classes, where a value of 1.0 is desirable. Once the classes were identified, associations between background characteristics and identified subclass were tested, including age, sex, well-being score, depressive symptoms score, anxiety score, psychological care, compliance of the app, and COVID-19 related items (including tested positive for COVID-19/or experience symptoms for COVID-19), and affection of a family member through COVID-19 (defines as being medically affected or financially affected).

To address the second aim, to understand the potential underlying mechanisms for coping, it was tested whether adolescents felt better on days with more adaptive coping, and whether they reported more positive affect on days that they did a challenge (i.e., a within-person fixed effect model). Finally, it was tested whether coping could explain within-person associations between challenges and positive affect (mediating effect through coping), by estimating causal mediation effects.

### Sensitivity analysis

In addition to these preregistered models, sensitivity analyses were conducted to test the robustness of our findings. As the age range of users was quite broad (12–25 years) age stratification (<16, >16 years) was performed to estimate the within-person associations with positive affect, negative affect, and coping. It was tested whether a significant difference in terms of engagement in the Grow It! app would exist between younger and older adolescents, including the measures for compliance of ESM surveys and CBT-based challenges.

### Missing Data Analysis

As in any intensive longitudinal study, missing data existed (van Roekel et al., 2019). However, Little’s MCAR test on the full data per cohort (i.e., daily positive affect, daily negative affect, daily adaptive coping) indicated that the pattern on missing data did not deviate from a MCAR pattern (cohort 1: Little’s MCAR test: χ2 = 0133, DF = 2, *p* = 0.936; cohort 2: χ2 = 2501, DF = 2, *p* = 0.286). Therefore, all available data was included in the models. A Student’s *t*-test was performed to determine whether there is a statistical difference between the baseline sample and the current sample, including age, sex, depressive symptoms, anxiety symptoms, and well-being. For cohort 1, the baseline sample was slightly older than our participants (16.7 years vs. 16.2 years, *p* < 0.01), and there were fewer girls on average at baseline compared to the individuals in the current study (67.6 vs. 76.1%, *p* < 0.001). No differences were observed in other variables. For cohort 2, there were no statistical differences between the baseline and the current sample. Finally, Student’s *t*-test was performed to test the difference between participants with available ESM data (cohort 1: *n* = 849, cohort 2: *n* = 1478) and the ones that surpass the ESM compliance and played CBT-based challenges criteria of the current study (cohort 1: *n* = 476, cohort 2: *n* = 814) (see Supplementary Tables 1-2). Only in the first cohort was sex significantly different between these two groups (*p* = 0.014), indicating that it is unlikely that bias was introduced by applying the exclusion criteria.

## Results

### Aim 1. Understanding Changes Every Day Well-being

#### Positive affect

Regarding changes in positive affect (Table 2), while controlling for COVID-19 stringency and daily weather (Model 3), a small but significant decrease in mean levels of positive affect over the course of the study was found in both cohorts (cohort 1: B = −0.09 per week, *p* < 0.0001, cohort 2: B = −0.11 per week, *p* < 0.0001), which did not confirm our hypotheses that participants would increase in daily positive affect over the course of the study.

**Table 2:** Results of the relation between the Grow It! app and daily positive affect

DV = Positive Affect	Cohort I			Cohort II		
	Model 1	Model 2	Model 3	Model 1	Model 2	Model 3
*Fixed effects*						
Intercept	4.73 (0.05)***	4.72 (0.05)***	4.72 (0.05)***	4.26 (0.04) ***	4.26 (0.04)***	4.26 (0.04)***
Week of study *Covariates (fixed)*		−0.09 (0.02)***	−0.09 (0.02)***		−0.11 (0.02)***	−0.11 (0.02)***
Daily COVID stringency			0.0005 (0.003)			0.03 (0.008)**
Daily COVID stringency × week of study			−0.001 (0.001)			−0.02 (0.008)**
Daily weather			0.0009 (0.0002)***			−0.0007 (0.0002)**
Daily weather × week of study			−0.0003 (0.0001)*			−0.00007 (0.0002)
*Random effects*						
Between person variance	1.27	1.21	1.21	1.13	1.14	1.13
Within person variance	0.92	0.84	0.84	0.99	0.92	0.92
Random effect variance around week of study		0.07	0.07		0.13	0.13
*Model*						
ICC	0.57	0.61	0.61	0.53	0.57	0.57
*N* individuals	476	476	476	814	814	814
*N* observation	23928	23928	23928	29607	29607	29607

Model 1: unconditional model. Model 2: unconditional growth model with notifications as random effect. Model 3: conditional growth model with fixed effects for daily COVID stringency and daily weather, random effects of notifications and interaction terms between COVID stringency*notifications and weather*notifications

*p < 0.05, **p < 0.01, ***p < 0.001

**Fig. 2:**
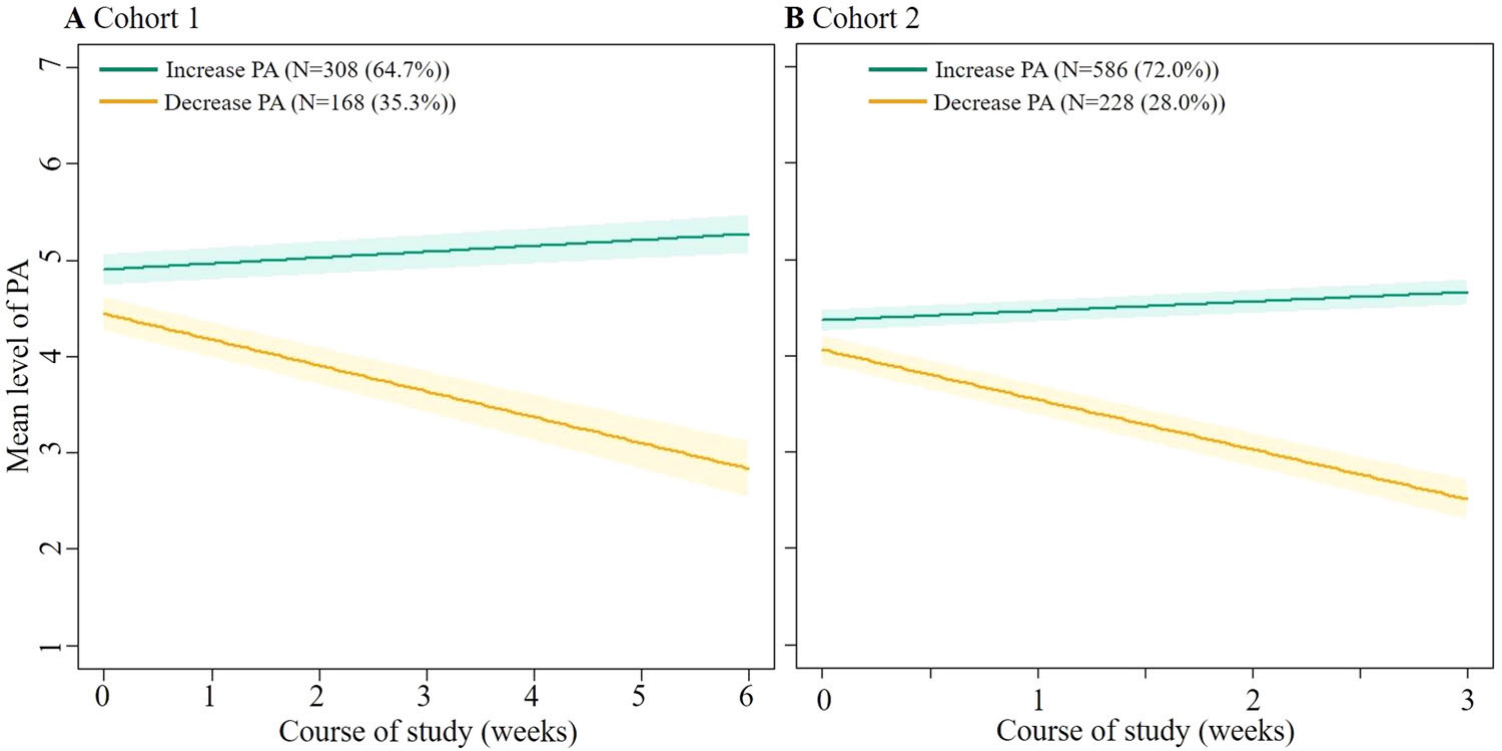
Observed mean levels of positive affect from two class-specific trajectories across two cohorts. Latent classes are presented for cohort 1 (**A**) and cohort 2 (**B**). The green line indicates an identified class increasing in positive affect. The orange line indicates a decrease in positive affect. We saw an increase in positive affect for 64.7% (*n* = 308) in cohort 1 and 72.0% (*n* = 586) in cohort 2. The decrease in positive affect class was represented by 35.3% (*n* = 168) in cohort 1 and 28.0% (*n* = 228) in cohort 2

**Fig. 3:**
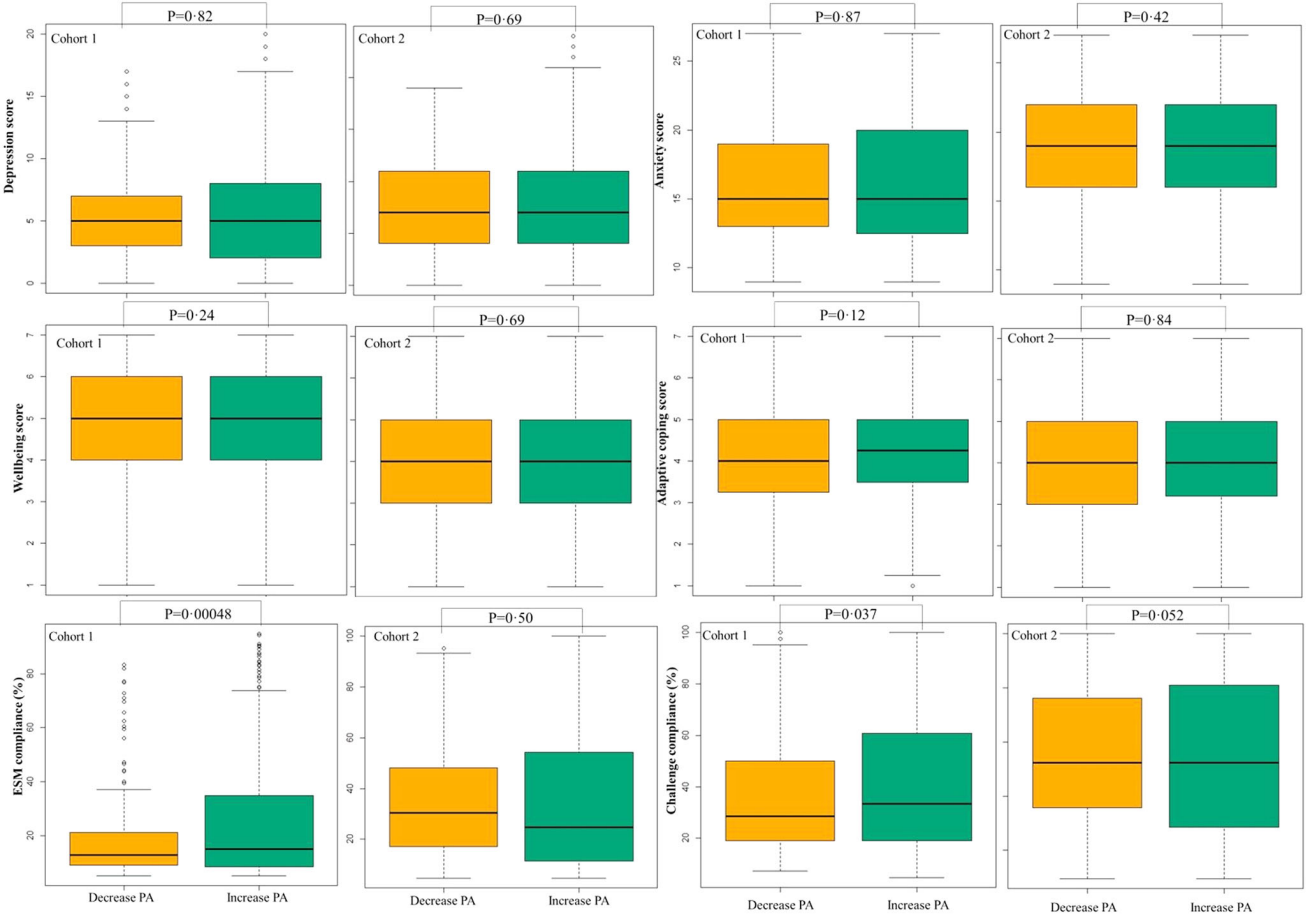
Boxplot illustrating differences between baseline characteristics of identified class-specific positive affect trajectories across two cohorts

To assess for how many adolescents positive affect would increase or decrease, differences between individuals in their positive affect trajectories were explored. Latent class growth models indicated that a 2-class model had a better fit (i.e., a lower Aikake and Bayesian information criterion), compared to a 1-class model (Table S3). This indicates significant heterogeneity in the population, and the presence of distinct subclasses. Most adolescents (cohort 1: *n* = 308 (64.7%); cohort 2: *n* = 586 (72.0%)) were classified as having a slight increase in positive affect over the course of the study as indicated by the estimated effect coefficients of 0.06 (*p* < 0.0001) for the first cohort and 0.10 (*P* < 0.001) for the second cohort. The other class (cohort 1: *n* = 168 (35.3%); cohort 2: *n* = 228 (28.0%)) was characterized by decreases in positive affect (Fig. 2), displayed by the estimated effect estimates of −0.27 (*p* < 0.0001) for the first cohort and −0.52 (*p* < 0.0001) for the second cohort. Background characteristics of the classes are shown in Fig. 3 and Table S4. Few differences were found. Participants in the group with an increase in positive affect in cohort 1 had higher ESM compliance and played more CBT-based challenges compared to the group that decreased in positive affect. In cohort 2 there were on average more boys with an increase in positive affect than girls.

#### Negative affect

Regarding negative affect, Table 3 (Model 3) demonstrates no changes in daily negative affect over the course of the six-week (cohort 1) and three-week (cohort 2) intervention, which ran counter to our expectation that participants would decrease in daily negative affect over the course of the study. Even though unconditional growth models (Model 2) indicated an increase of 0.04 units in negative affect per week from Grow It! for cohort 1, this could be explained by the increasing COVID-19 stringency and decreasing daily weather.

**Table 3:** Results of the relationship between the Grow It! app and daily negative affect

DV = Negative affect	Cohort I			Cohort II		
	Model 1	Model 2	Model 3	Model 1	Model 2	Model 3
*Fixed effects*						
Intercept	1.88 (0.04)***	1.88 (0.04)***	1.88 (0.04)***	2.07 (0.03)***	2.07 (0.03)***	2.07 (0.03)***
Week of study		0.03 (0.01)***	0.02 (0.01)		0.03 (0.02)	0.03 (0.02)
*Covariates (fixed)*						
Daily COVID stringency			−0.006 (0.002)**			−0.03 (0.007)***
Daily COVID stringency × week of study		−0.00009 (0.0008)			0.02 (0.007)**	
Daily weather			−0.0001 (0.0001)			0.0004 (0.0002)*
Daily weather × week of study			0.0002 (0.0001)*			0.0006 (0.0002)
*Random effects*						
Between person variance	0.57	0.57	0.57	0.63	0.63	0.63
Within person variance	0.59	0.55	0.55	0.68	0.62	0.62
Random effect variance around week of study		0.03	0.03		0.12	0.12
*Model*						
ICC	0.49	0.53	0.54	0.48	0.53	0.53
N individuals	476	476	476	814	814	814
N observation	23928	23928	23928	29607	29607	29607

Model 1: unconditional model. Model 2: unconditional growth model with Grow It app as random effect. Model 3: conditional growth model with fixed effects for daily COVID stringency and daily weather, random effects of Notifications and interaction terms between COVID stringency*notifications and weather*notifications

*p < 0.05, **p < 0.01, ***p < 0.001

**Fig. 4:**
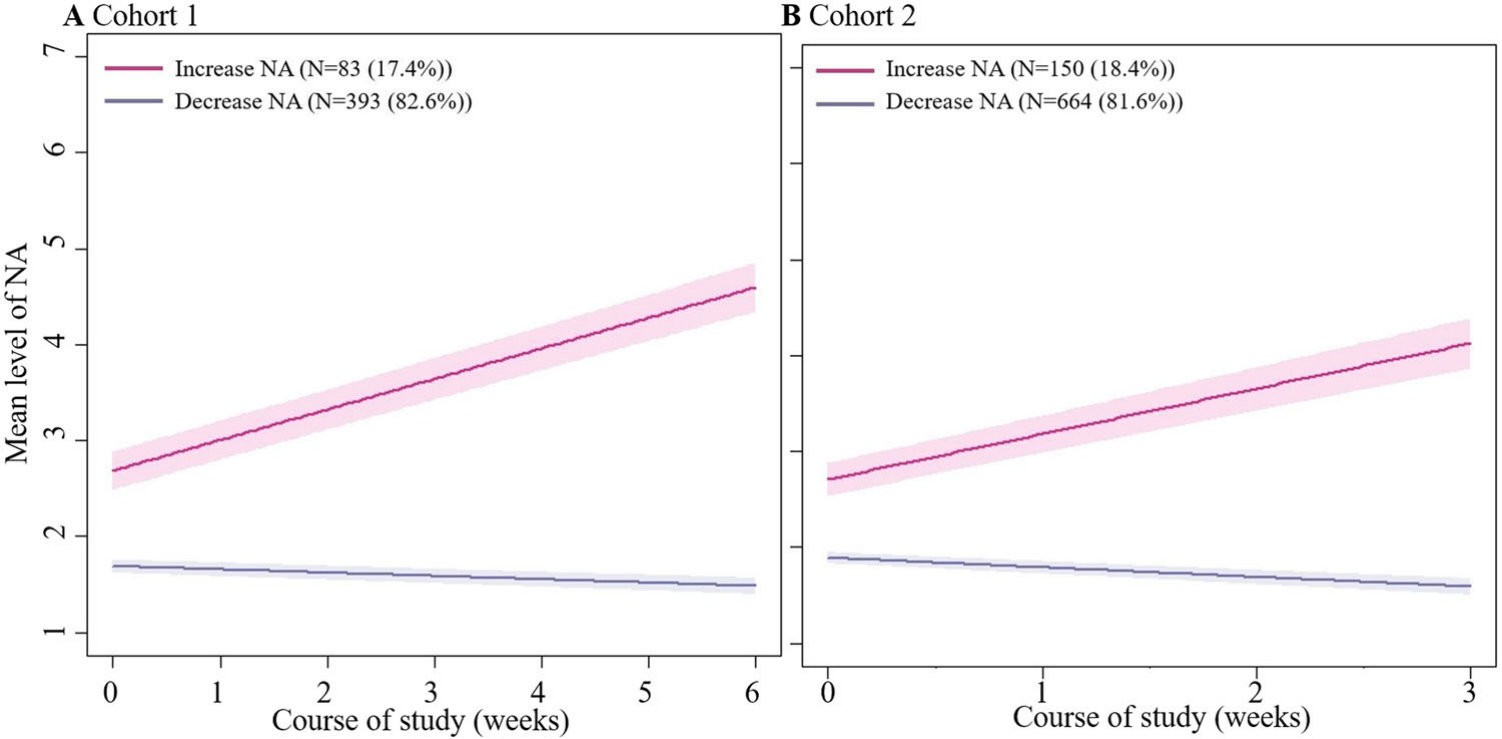
Observed mean levels of negative affect from two class-specific trajectories across two cohorts. Latent classes are presented for cohort 1 (**A**) and cohort 2 (**B**). The purple line indicates an identified class decrease in negative affect. The pink line indicates an increase in negative affect. We saw an increase in negative affect for 17.4% (*n* = 83) in cohort 1 and 18.4% (*n* = 150) in cohort 2. The decrease in negative affect class was represented by 82.6% (*n* = 393) in cohort 1 and 81.6% (*n* = 664) in cohort 2

**Fig. 5:**
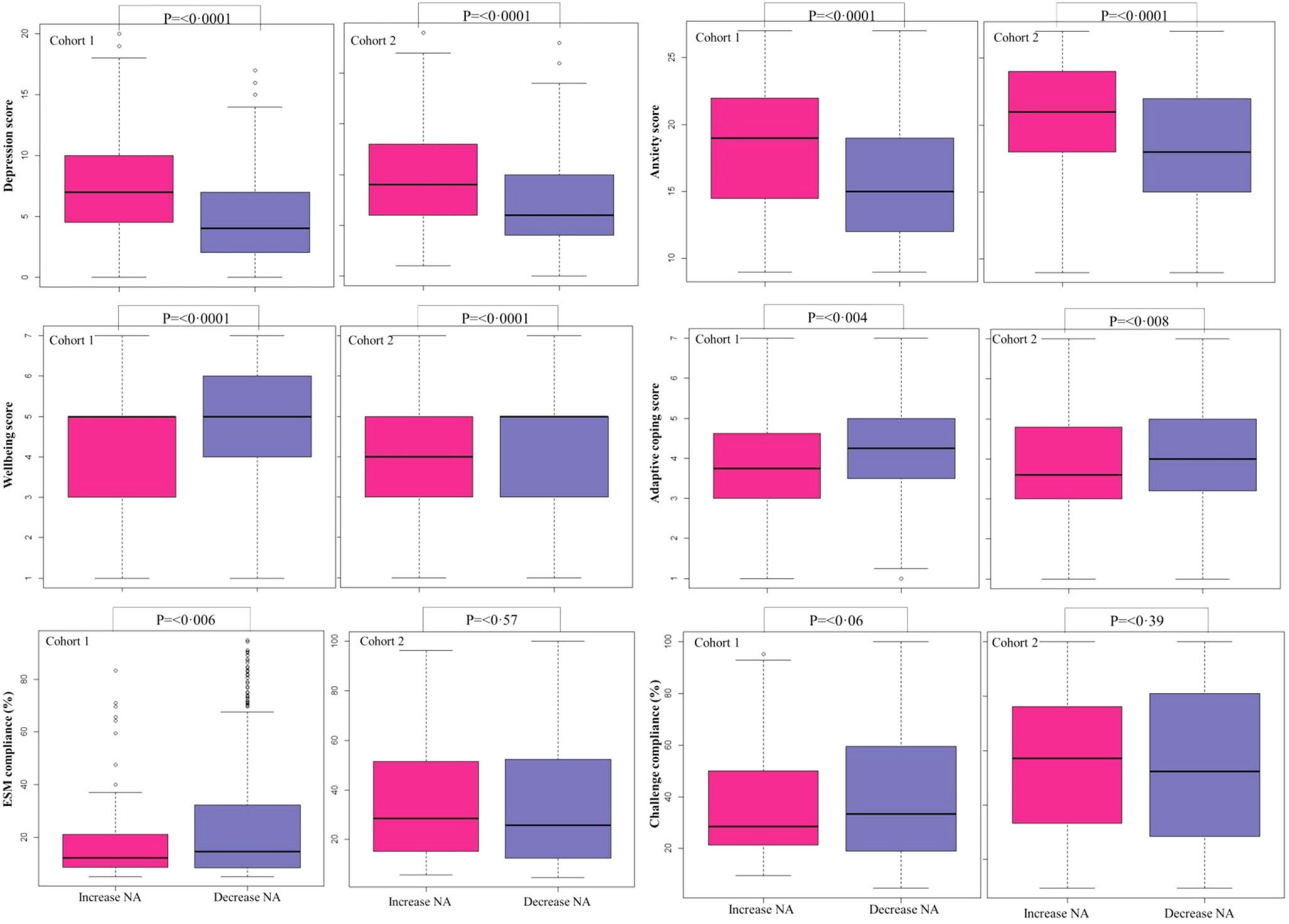
Boxplot illustrating differences between baseline characteristics of identified class-specific negative affect trajectories across two cohorts

Again, exploring effect heterogeneity between individuals in their negative affect trajectory, two unique classes were identified (Table S5). The largest class was characterized by a decreasing negative affect over the course of the study (cohort 1: *n* = 393 (82.6%); cohort 2: *n* = 664 (81.6%)). Given the estimated effect coefficients, of −0.03 (*p* < 0.0001) for the first cohort and −0.10 (*p* < 0.0001) for the second cohort, most individuals within this class display a significant decrease in negative affect while playing the Grow It! app. The other less prevalent class (cohort 1: *n* = 83 (17.4%); cohort 2: *n* = 150 (18.4%)) showed a significant increase in daily negative affect, with an estimated effect coefficient of 0.32 (*p* < 0.0001) in the first cohort and 0.47 (*p* < 0.0001) in the second cohort (Fig. 4). Participants in the class who reported an increase in daily negative affect had significantly higher levels of depressive symptoms and anxiety and significantly lower levels of well-being and adaptive coping at baseline than in the class following a decrease in daily negative affect (Fig. 5 and Table S6). Also, the class with an increase in daily negative affect were more often in treatment for psychological care (*n* = 57 (38.0%)) vs *n* = 115 (17.3%)) and had significantly lower ESM compliance in the first cohort (18.8% vs. 25.0%). In other words, adolescents who improved most in their well-being over the course of the study were characterized by lower levels of psychological problems, and were more engaged in terms of their ESM compliance.

### Aim 2. Understanding the Mechanisms. Adaptive Coping and CBT-based Challenges

Grow it contains CBT-based challenges, which aim to promote more adaptive cooping. However, as indicated in Table 4 (Model 3), adolescents’ adaptive coping decreased over the course of the study for both cohorts in the conditional growth model (cohort 1: B = −0.12 per week, *p* < 0.0001, cohort 2: B = −0.12 per week, *p* < 0.0001).

**Table 4:** Results of the association between adaptive coping and course of study

DV = adaptive coping	Cohort I			Cohort II		
	Model 1	Model 2	Model 3	Model 1	Model 2	Model 3
*Fixed effects*						
Intercept	3.85 (0.05)***	3.85 (0.05)***	3.84 (0.05)***	3.81 (0.03)***	3.81 (0.03) ***	3.81 (0.03)***
Week of study		−0.08 (0.02)***	−0.12 (0.03)***		−0.12 (0.02) ***	−0.12 (0.02)***
*Covariates (fixed)*						
Daily COVID stringency			−0.01 (0.007)			−0.009 (0.02)
Daily COVID stringency × week of study			−0.002 (0.003)			−0.04 (0.02)
Daily weather			−0.0004 (0.0005)			0.0001 (0.0005)
Daily weather × week of study			0.0008 (0.0004)*			0.0002 (0.0005)
*Random effects*						
Between person variance	0.80	0.81	0.82	0.62	0.63	0.63
Within person variance	1.42	1.38	1.37	1.15	1.08	1.08
Random effect variance around week of study		0.02	0.02		0.09	0.09
*Model*						
ICC	0.36	0.38	0.38	0.35	0.39	0.38
*N* individuals	468	468	468	779	779	779
*N* observation	4654	4654	4654	5892	5892	5892

Model 1: unconditional model. Model 2: unconditional growth model with notifications as random effect. Model 3: conditional growth model with fixed effects for daily COVID stringency and daily weather, random effects of notifications and interaction terms between COVID stringency*notifications and weather*notifications

*p < 0.05, **p < 0.01, ***p < 0.001

At the same time, a positive association was found between within-person changes in adaptive coping and within-person changes in positive affect (cohort 1: B = 0.16, *p* < 0.0001, cohort 2: B = 0.17, *p* < 0.0001). This indicates that on days when individuals employ more coping strategies (than they normally would), they experienced a more positive affective well-being compared to other days and vice versa. However, whether individuals had played a challenge that day, was independent to their positive affect (cohort 1: B = 0.002, *p* = 0.93, cohort 2: B = 0.07, *p* = 0.54), nor with adaptive coping (cohort 1: B = −0.054, *p* = 0.44; cohort 2: B = 0.0004, *p* = 0.99). Taking into account the baseline coping levels, the association between playing a challenge and daily adaptive coping remained statistically insignificant. Following the criteria for a variable to be defined as a mediator (e.g., exposure significantly affects outcome), it was not able to test the hypothesized meditative association between daily CBT-based challenges on positive affect via within-person changes in adaptive coping.

### Sensitivity Analyses

Because the sample had a large age range, sensitivity analyses were conducted with two distinct age groups. Younger adolescents were defined as younger than 16 years, whereas older adolescents were defined as 16 years or older. In cohort 1, there were 237 participants older than 16 years (49.8% of the ESM sample). In the first cohort, 20.6% of Grow It! users were enrolled in college. In cohort 2, there were 613 participants 16 years or older (75.3% of the ESM sample). In the second cohort, 49.5% of the Grow It! users were enrolled in college. In cohort 1, younger participants completed on average less ESM surveys compared to the older participants (ESM responses: younger: average 41.4 ESM, older: 59.2 ESM, *p* < 0.0001), however there was no difference observed in the number of played challenges (younger: average: 16.4 challenges, older: 17.9 challenges, *p* = 0.17).In cohort 2, younger participants had an average of 33.4 ESM responses against 37.2 ESM in the older group. Based on the Student’s *t*-test this was not significantly different (*p* = 0.078). Similarly, there was no significant difference in the engagement of played challenges (younger: average 11.9 challenges, older: 11.2 challenges, *p* = 0.18).

Furthermore, it was tested whether the distinct age groups experienced differences in benefits of the app. Regarding daily positive affect, analysis revealed that younger participants decreased in mean levels of positive affect (cohort 1: B = −0.101, *p* < 0.0001, cohort 2: B = −0.152, *p* < 0.0001), as well as older participants (cohort 1: B = −0.09, *p* < 0.0001, cohort 2: −0.102, *p* < 0.0001). For negative affect, analysis revealed that younger participants significantly increased in mean levels of negative affect (cohort 1: B = 0.04, *p* = 0.02, cohort 2: 0.093, *p* < 0.01), whereas older participants demonstrated no significant change in mean levels of negative affect (cohort 1: B = 0.012, *p* = 0.51, cohort 2: B = 0.02, *p* = 0.27). For adaptive coping, we found that younger participants experienced a decrease in mean levels of adaptive coping in both cohorts (cohort 1: B = −0.311, *p* < 0.0001, cohort 2: B = −0.17, *p* < 0.05), and older participants experienced decrease in mean levels of adaptive coping in cohort 2 only (cohort 1: B = −0.343, *p* = 0.33, cohort 2: −0.11, *p* < 0.0001).

## Discussion

Adolescence is a critical period for psychological and social changes, with important implications for emotional well-being, both immediately and later in life (Ciranka & van den Bos, 2019). Important elements for young people to move through adolescence smoothly include involvement in social interactions, autonomy, and identity development. Nevertheless, adolescents worldwide are experiencing a decline in their emotional well-being, which is reflected in an increase in depressive and anxiety symptoms, and this situation has even worsened due to the COVID-19 pandemic (Ma et al., 2021). The current healthcare system cannot meet the demand of people seeking psychological care (American Psychological Association, 2020). mHealth applications has the potential to provide mental health support in a very accessible way. Even though the Grow it! app aimed at promoting well-being and coping by integrating CBT-based challenges has shown promising results in improving overall well-being (Dietvorst, Aukes, et al., 2022; Dietvorst et al. 2022); daily changes in positive and negative affect and adaptive coping had not been studied. The present study therefore examined the daily changes in positive and negative affect and coping using ESM, assessed whether some individuals may benefit more than others, and examined to which extent this mechanism can be explained by “playing” CBT-based challenges.

The results showed that, on average, adolescents decreased in daily positive affect and adaptive coping, and increased in negative affect. Yet 64.7–72.0% of adolescents experienced the expected increase in positive affect and 81.6–82.6% a decrease in negative affect, and these adolescents were characterized by fewer depressive and anxiety symptoms and more ESM- involvement. As a first indication of the potential of promoting coping, a positive relation between adaptive coping and positive affect was found. This indicates that an improved ability to cope with stressful events associates with improvements in daily affective well-being; however, this observation could not be explained by the number of CBT-based challenges played. These key-findings are discussed in more detail below.

### Heterogeneous Trajectories of Changes in Positive and Negative affect

In this study, changes in everyday positive and negative affect were studied among adolescents who played the Grow It app. The theory behind positive and negative affect is that they reflect emotional experience in an independent way of each other when measured through the PANAS (Watson et al., 1988). Individuals can experience both high and low levels of positive affect and negative affect simultaneously; however, the extent to which these measures are opposite dimensions remains a matter of debate (Barrett & Russell, 1998). In this study, both were assessed, and the findings point at similar insights.

On average, a decline was observed in how much positive affect adolescents experienced during the study. However, the results of the current study also suggest that 64.7–72.0% of the users increase in positive affect. Although the baseline character of the group with an increase in positive affect versus the group with a decrease in positive affect was similar, the few observed differences that emerged were that girls were more likely to have a decrease in positive affect (cohort 2), and those with higher adherence in ESM and challenges increased in positive affect (cohort 1). However, the low entropy score (0.41 and 0.54) indicates that the model did not fit optimally and these findings should be interpreted with caution.

Even though on average negative affect increased, 81.6–82.6% of the Grow It! users were classified into a trajectory of decreasing negative affect. Several significant baseline predictors that could partly explain the identified clusters and were associated with a decrease in negative affect, including being a boy (cohort 2), lower depression and anxiety scores, higher affective well-being score, higher coping score, receiving less psychological care (cohort 2), experience less COVID-19 related symptoms (cohort 2), higher ESM compliance (cohort 1). The rest (~18% of users) followed a sharp decline in daily well-being. Given the severity of their emotional problems and care use at baseline, this more seriously affected group seems as expected to benefit less from the prevention-oriented Grow It! app and may need more embedded clinical support. Because of the COVID-19 related necessary recruitment strategy, minimal exclusion criteria were applied.

The observed average decline in both positive affect and overall increase in negative affect can be explained in two ways. Firstly, the finding is in accordance with other COVID-19 studies examining well-being of adolescents. For example, across the globe, depression and anxiety disorders have increased up to 30% due to COVID-19(age range 9-99 years) (Santomauro, 2021). Another study researching well-being of Canadian children and adolescents (*n* = 932) found that nearly half of their sample reported changes that could contribute to lower well-being (Mitra et al., 2021). Therefore, the more surprising finding of the current study is not that 18–35% reported worse daily functioning, but that 65–82% improved in the opposite direction and may actually benefit from the effects of the Grow It! app. Secondly, as earlier work on this app showed an average increase in well-being and decrease in depressive symptoms (Dietvorst et al. 2022), it may also be that players become more self-aware (due to the ESM monitoring) of a wider range of their emotions, rather than an actually decrease in their well-being. Future research will need to examine these potential explanations.

### Coping as Underlying Mechanism for Improvements in Well-being

The second aim of this study was to better understand the mechanisms behind changes in well-being and internalizing symptoms. Increases in daily well-being among most players of the Grow It! app may be the result of two mechanisms; through increasing self-insight in emotions and promoting resilience as a result of adaptive coping. To this end, the Grow It! app focuses on strengthening a variety of coping strategies such as seeking social support, self-acceptance, positive distractions, and problem solving. These coping skills are key elements of building resilience (Masten et al., 2021), which in turn is positively associated with well-being.

Since coping strategies are an important resilience factor (Mesman et al., 2021) that may help adolescents to feel positive despite external stressful circumstances, the within-person association was examined between adaptive coping and positive affect. Although the study design does not allow testing for a causal association, this study found that on days when individuals applied more adaptive coping than they typically do, they also reported more positive affect; however, no positive effect was found of the CBT-based challenges on either positive affect or adaptive coping. Furthermore, findings of this study indicate that on average participants decreased on how they perceived the strength of their own coping strategies during the study. As with their improved emotional awareness, a plausible explanation for this is that the participants became more aware of their applied coping strategies through Grow It!. In addition, as government restrictions were still in place, the CBT-based challenges had to be modified and could not focus entirely on aspects such as social support including physical activation (e.g., participants were unable to see friends in person and relied more on online contact). Limited social support from peers (Pouwels et al., 2021) may also have influenced adolescents’ own perception of how active their coping was.

Since everyone’s ability to cope with stressful events has been put to the test, all adolescents are considered at risk of declining well-being (Clemens et al., 2020). Furthermore, times of stressful events are linked to a growing polarization in the ability for adolescents to develop (Masten & Motti-Stefanidi, 2020). A possible explanation for this is that the individual’s ability to cope with stressful events varies from person to person and unprecedented events can divert this difference (Masten & Motti-Stefanidi, 2020). The results of this study highlight that by observing that individuals with less coping skills at baseline are more likely to further decline in well-being and benefit less from interventions designed for the general population. Previous efforts in developmental research show that through periodically reporting emotions using ESM, self-awareness improves; however, this mechanism is better developed in older adolescents (Heller & Casey, 2016). In theory, therefore, mHealth and CBT-based interventions may be more beneficial for older adolescents than younger adolescents. Somewhat unexpectedly, no substantial differences were observed in the present study in terms of both positive affect and adaptive coping. Only for negative affect a significant increase was observed in younger adolescents, while this was not the case in older adolescents. Future studies, showing higher compliance in both ESM and CBT-based challenges, are needed to further investigate an age-dependent mechanism.

### Mental Health During COVID-19 Lockdowns

In this study, two groups of youth were followed for six weeks during the first lockdown and three weeks during the second lockdown. Although, as can be seen in Table 1, participants in the second cohort scored at baseline worse on depressive symptoms, anxiety, affective well-being, and average levels of positive and negative affect compared to those in the first cohort. This finding mimics what other scholars have shown, that the effects of the COVID-pandemic may last and even worsen over the course of repeated lock-downs (Loades et al., 2020). In addition, the impact of COVID-19 was greater during the second lockdown, as evidenced by the magnitude effect on all three outcome variables. Furthermore, users in cohort 2 reported that if they were experiencing COVID-19 related symptoms they were more likely to increase in negative affect. One of the main differences between the first and second lockdown is the curfew. Contrary to what we expected that older adolescents might have been harder hit by the curfew, no significant difference was observed in effect on daily well-being between age groups in the second lockdown versus the first. However, adolescents in the second cohort did not experience a stronger decline in positive affect or adaptive coping over the course of the study (Tables 2 and 4).

Altogether, it is difficult to make hard assumptions about differences/similarities from the two cohorts, for several reasons. For example, it is almost impossible to see the crude effect of the Grow It! app against external factors, including COVID-19 measures. In addition, although the recruitment strategy for both cohorts was similar, the study participants in both cohorts are different. Participants in the second cohort had been dealing with stressful events for a longer period of time, and both their intention to use the app as well as the perceived effect of an mHealth intervention may differ.

### Limitations and Strengths

Even though this intensive longitudinal study among large samples used a preregistered plan of analyses, and two independent cohorts, some limitations need to be considered. The use of mHealth is not equally attractive to all adolescents, as some are particularly drawn to it. As such, the generalizability of the current findings should be explored in a future study with better national representation, and within other nations, as the sample of the current study is homogeneous in terms of background characteristics (i.e., more girls, highly educated, Dutch ethical background). Furthermore, highly motivated individuals are especially willing to participate in such research. This suggestion is confirmed because only less than half of the subjects who completed the baseline questionnaire were included in the current study. Nevertheless, missing data was at random and we saw no difference in baseline characteristics between participants excluded due to low compliance and those included (Table S1 and Table S2), indicating that there was no evidence for induced selection bias. Furthermore, the Grow It! app is a study with scientific research purposes, including an extensive informed consent procedure, which raises a threshold for participation. In addition, the compliance of this ESM study was lower compared to other scientific studies in adolescent samples (van Roekel et al., 2019). One way of increasing compliance is to pay adolescents for their participation (van Roekel et al., 2019), but as our aim was to test the effectiveness of this app in real settings, this strategy was not chosen. In other online youth interventions, these compliance rates are comparable (Linardon & Fuller-Tyszkiewicz, 2020). In the future, where mHealth becomes more commonly integrated in healthcare, designs need to be tailored to the individual and personal feedback is needed. Another limitation is the absence of pre-COVID-19 data and a control group. We were therefore unable to test whether the observed effect can be attributed to Grow It! for users compared to non-users. Instead, we adjusted for COVID-19 measures in our analysis by using the stringency index. Also, we could not investigate the effect in relation to a non-pandemic situation. Notwithstanding these limitations, this large-scale microscopic study of adolescent’s well-being in daily lives lays the groundwork for future research into gamified smartphone tools to promote adolescent well-being and coping.

## Conclusion

Previous studies have suggested that the Grow It! app is beneficial in promoting overall well-being by integrating CBT-based challenges in adolescents at the population level (Dietvorst et al. 2022). Using ESM, the present intense longitudinal study aimed to elucidate daily changes in positive affect, negative affect, and adaptive coping, and to investigate to what extent this mechanism can be explained by CBT-based challenges. This ESM study found that the majority of Grow It! app users improved in daily well-being. However, a small group of those who were at higher risk of developing mental health problems before the study started had significant declines in daily well-being and may need more embedded clinical support. As for the underlying mechanism, an within-person link between adaptive coping and positive affect suggests that coping helps adolescents be resilient in stressful times, but that this effect is unrelated to the CBT-based challenges. While continued efforts are needed to verify that the Grow It! app is effective as a prevention tool for adolescents in the general population, to identify and support populations at risk, and who may need an improved or different (more intensive) intervention, it has a potential preventive effect on the mental health of a subgroup of users during a stressful period in life. The latter has been demonstrated by the identified heterogeneous trajectories for both positive and negative affect, showing that participants with lower levels of psychopathology and who were more engaged in the ESM module were more likely to benefit from the Grow It! app.

## Supplementary information

Supplementary information
