# Supplementary material for: Promoting Daily Well-being in Adolescents using mHealth
Source: J Youth Adolesc. 2022 Jul 22;51(11):2173–89. doi: 10.1007/s10964-022-01656-8 (PMC9306228; doi:10.1007/s10964-022-01656-8)
Supplement: Supplementary file 1 — Supplementary information [file 10964_2022_1656_MOESM1_ESM.docx]

**Supplementary material**

Supplementary figure 1. Flowchart of the population for analysis.

Supplementary table 1. Baseline characteristics compared between participants included in current study population; cohort 1

Supplementary table 2. Baseline characteristics compared between participants included in current study population; cohort 2

Supplementary table 3. Fit indices for class model solutions positive affect trajectories

Supplementary table 4. Baseline characteristics stratified by identified latent classes on positive affect

Supplementary table 5. Fit indices for class model solutions negative affect trajectories

Supplementary table 6. Baseline characteristics stratified by identified latent classes on negative affect


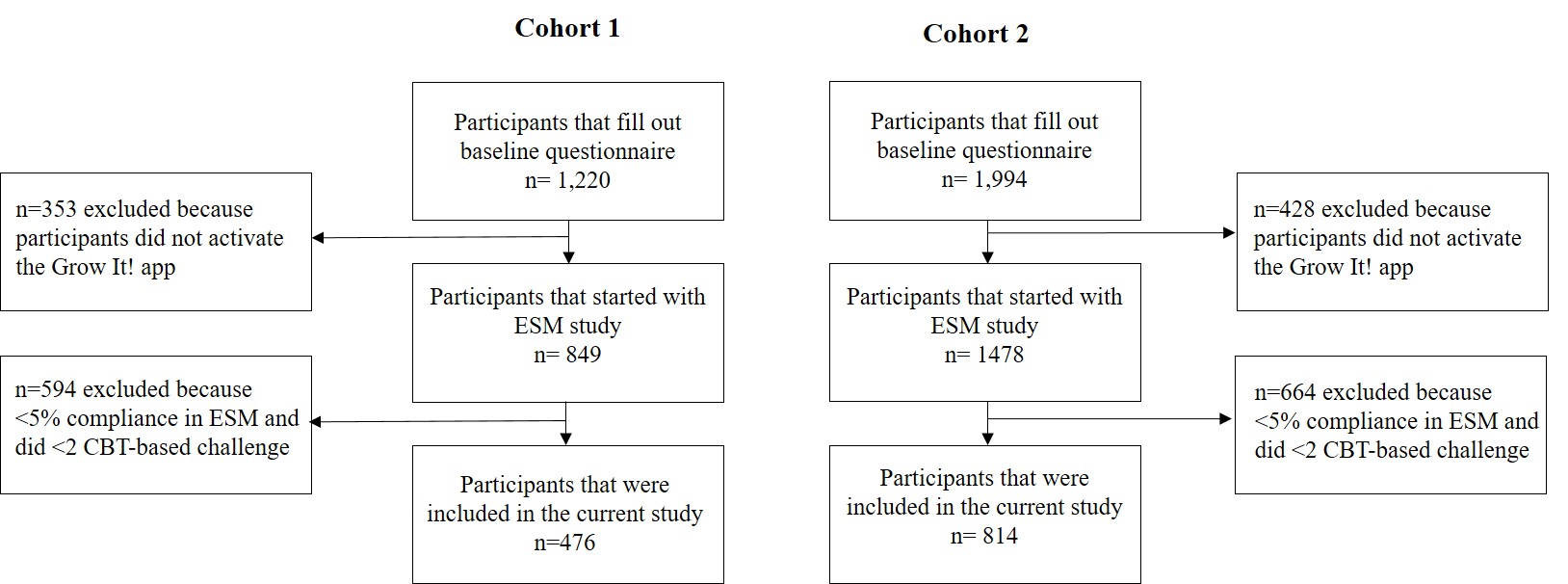


Supplementary figure 1. Flowchart of the population for analysis.

Supplementary table 1. Baseline characteristics compared between participants included in current study population; cohort 1

| Cohort 1 | Participants included in study  (n=476) | Study population (n=849) |  | P value of group difference |  |
| --- | --- | --- | --- | --- | --- |
| Age (years), mean (SD) | 16.24 (3.01) | 16.52 (3.30) |  | 0.12 |  |
| Female, n(%) | 362 (76.1%) | 596 (70.2%) |  | 0.014 |  |
| Depression (score), mean (SD) | 5.41 (3.98) | 5.56 (4.09) |  | 0.52 |  |
| Anxiety (score), mean (SD) | 16.24 (4.67) | 16.30 (4.85) |  | 0.81 |  |
| Wellbeing (score), mean (SD) | 4.92 (1.28) | 4.89 (1.40) |  | 0.69 |  |
| Adaptive coping (score), mean (SD) Psychological care    *Yes  Waiting list* | 4.23 (1.33)  65 (13.7%) 10 (2.1%) | 4.24 (1.26)  127 (15.0%) 22 (2.6%) |  | 0.94 0.72 |  |

Variables are expressed as mean (SD), or percentage (%). Difference between two cohorts based on Student t test.

Supplementary table 2. Baseline characteristics compared between participants included in current study population; cohort 2

| Cohort 2 | Participants included in study (n=814) | Study population (n=1478) |  | P value of group difference |  |
| --- | --- | --- | --- | --- | --- |
| Age (years), mean (SD) | 18.45 (3.44) | 18.65 (3.65) |  | 0.22 |  |
| Female, n(%) | 671 (82.8%) | 1178 (79.7%) |  | 0.15 |  |
| Depression (score), mean (SD) | 7.56 (4.47) | 7.36 (4.22) |  | 0.24 |  |
| Anxiety (score), mean (SD) | 18.91 (4.43) | 18.71 (4.48) |  | 0.27 |  |
| Wellbeing (score), mean (SD) | 4.26 (1.40) | 4.27 (1.41) |  | 0.77 |  |
| Adaptive coping (score), mean (SD) Psychological care    *Yes  Waiting list* | 4.04 (1.26)  172 (21.1%) 40 (4.9%) | 4.01 (0.99)  306 (20.7%) 67 (4.5%) |  | 0.94 0.98 |  |

Variables are expressed as mean (SD), or percentage (%). Difference between two cohorts based on Student t test.

|  |  | G | loglik | AIC | BIC | SABIC | entropy | ICL | %class1 (mean posterior probability) | %class2 (mean posterior probability) |  |
| --- | --- | --- | --- | --- | --- | --- | --- | --- | --- | --- | --- |
| Cohort 1 |  | 1 | -33738.13 | 67488.25 | 67513.24 | 70113.65 | 1 | 70132.70 | 100 |  |  |
|  |  | 2 | -34676.88 | 69373.75 | 69417.62 | 69385.87 | 0.41 | 68484.54 | 69.19 (0.79) | 30.81 (0.77) |  |
| Cohort 2 |  | 1 | -43200.18 | 86412.37 | 86440.58 | 86421.53 | 1 | 86440.58 | 100 |  |  |
|  |  | 2 | -42766.51 | 85553.02 | 85600.04 | 85568.29 | 0.54 | 84226.71 | 71.99 (0.85) | 28.01 (0.83) |  |

Supplementary table 3. Fit indices for class model solutions positive affect trajectories.

Akaike Information Criteria (AIC), Bayesian Information Criteria (BIC), and sample size adjusted Bayesian Information Criteria (ssaBIC), integrated complete-data likelihood criterion (ICL)

Supplementary table 4. Baseline characteristics stratified by identified latent classes on positive affect

|  | Cohort 1 |  |  | Cohort 2 |  | | |
| --- | --- | --- | --- | --- | --- | --- | --- |
|  | Increase positive affect (N=308, 64.7%) | Decrease positive affect (N=168, 35.3%) | P value of group difference | Increase positive affect (N=586, 72.0%) | Decrease positive affect (N=228, 28.0%) | P value of group difference |  |
| Intercept | 4.90 | 4.45 | <0.0001 | 4.37 | 4.06 | <0.0001 |  |
| Slope | 0.06 | -0.27 | <0.0001 | 0.10 | -0.52 | <0.0001 |  |
| Age (years) | 16.17 | 16.37 | 0.49 | 18.44 | 18.67 | 0.28 |  |
| Female Ethnicity   *Dutch  Non-Dutch  Mixed* Education   *primary school  secondary school  low  middle   high   university/college  low  middle  high   other* COVID positive/symptoms  Family member affected | 234 (76.0%)  272 (88.3%) 2 (0.6%) 25 (8.1%)  19 (6.2%)  19 (6.2%) 45 (14.6%) 159 (51.6%)  21 (6.8%) 16 (5.2%) 23 (7.5%) 1 (0.3%) 24 (7.8%) 34 (11.0%) | 128 (76.2%)  150 (89.3%) 0 (0%) 10 (6.0%)  8 (4.8%)  11 (6.5%) 25 (14.9%) 82 (48.8%)  10 (5.9%) 16 (9.5%) 12 (7.1%) 1 (0.6%) 18 (10.7%) 20 (11.9%) | 0.96 0.17               0.79 0.55 | 473 (80.1%)  571 (97.4%) 1 (0.2%) 11 (1.9%)  7 (1.2%)  33 (5.6%) 82 (14.0%) 124 (21.2%)  64 (10.9%) 99 (16.9%) 126 (21.5%) 32 (5.5%) 75 (12.8%) 108 (18.4%) | 198(87.2%)  220 (96.5%) 1 (0.4%) 5 (2.2%)  0 (0%)  15 (6.6%) 28 (12.3%) 49 (21.5%)  25 (11.0%) 47 (20.6%) 42 (18.4%) 18 (7.9%) 32 (14.0%) 45 (19.7%) | 0.021 0.64  0.78 0.65 |  |
| Depression | 5.38 | 5.47 | 0.82 | 7.63 | 7.49 | 0.69 |  |
| Anxiety | 16.26 | 16.19 | 0.87 | 18.85 | 19.13 | 0.42 |  |
| Well-being | 4.97 | 4.82 | 0.24 | 4.27 | 4.23 | 0.69 |  |
| Adaptive coping  Psychological care  *Yes   Waiting list* | 4.30  48 (15.6%) 6 (1.9%) | 4.10  17 (10.1%) 4 (2.4%) | 0.12  0.11 | 4.03  121 (20.6%) 27 (4.6%) | 4.05  51 (22.4%) 13 (5.7%) | 0.84 0.38 |  |
| ESM | 55.32 | 41.00 | 0.00047 | 35.98 | 37.32 | 0.50 |  |
| Challenge | 17.91 | 15.65 | 0.037 | 11.18 | 12.04 | 0.05 |  |

Supplementary table 5. Fit indices for class model solutions negative affect trajectories.

|  | G | loglik | AIC | BIC | SABIC | entropy | ICL | %class1 (mean posterior probability) | %class2 (mean posterior probability) |
| --- | --- | --- | --- | --- | --- | --- | --- | --- | --- |
| Cohort 1 | 1 | -29619.35 | 59250.70 | 59277.02 | 59257.97 | 1 | 59277.02 | 100 |  |
|  | 2 | -29212.59 | 58445.18 | 58489.05 | 58457.30 | 0.72 | 57391.50 | 17.4 (0.87) | 82.83 (0.95) |
| Cohort 2 | 1 | -37590.48 | 75192.95 | 75221.16 | 75202.11 | 1 | 75221.16 | 100 |  |
|  | 2 | -37085.30 | 74190.60 | 74237.62 | 74205.87 | 0.70 | 72745.14 | 18.43 (0.87) | 81.57 (0.93) |

Akaike Information Criteria (AIC), Bayesian Information Criteria (BIC), and sample size adjusted Bayesian Information Criteria (ssaBIC), integrated complete-data likelihood criterion (ICL)

Supplementary table 6. Baseline characteristics stratified by identified latent classes on negative affect

|  | Cohort 1 |  |  | Cohort 2 |  | |  |
| --- | --- | --- | --- | --- | --- | --- | --- |
|  | Increase negative affect  (N=83, 17.4%) | Decrease negative affect  (N=393, 82.6%) | P value | Increase negative affect (N=150, 18.4%) | Decrease negative affect  (N=664, 81.6%) | | P value |
| Intercept | 2.69 | 1.68 | <0.0001 | 2.71 | 1.89 | <0.0001 | |
| Slope | 0.32 | -0.03 | <0.0001 | 0.47 | -0.10 | <0.0001 | |
| AGE | 16.51 | 16.18 | 0.38 | 18.43 | 18.47 | 0.90 | |
| Female  Ethnicity   *Dutch  Non-Dutch  Mixed* Education   *primary school  secondary school  low  middle   high   university/college  low  middle  high   other* COVID positive/symptoms  Family member affected | 66 (79.5%)  75 (90.4%) 0 (0%) 6 (7.2%)  3 (3.6%)  3 (3.6%) 13 (15.7%) 40 (48.2%)  5 (6.0%) 8 (9.6%) 2 (2.4%) 1 (1.2%) 12 (14.5%) 11 (13.3%) | 296 (75.3%)  347 (95.2%) 2 (0.5%) 29 (7.4%)  6 (1.5%)  27 (6.9%) 57 (14.5% ) 201(51.1%)  26 (6.6%) 24 (6.1%) 33 (8.4%) 1 (0.3%) 30 (7.6%) 43 (10.9%) | 0.42 0.87               0.14 0.49 | 133 (88.7%)  145 (96.7%) 0 (0%) 5 (3.3%)  0 (0%)  12 (8.0%) 24 (16.0%) 33 (22.0%)  9 (6.0%) 27 (18.0%) 17 (11.3%) 15 (10.0%) 28 (18.7%) 32 (21.3%) | 538 (81.0%)  646 (97.3%)  2 (0.3%) 11 (1.7%)  7 (1.1%)  36 (5.4%) 86 (13.0%) 140 (18.1%)  69 (10.4%) 119 (17.9%) 151 (22.7%) 35 (5.3%) 79 (11.9%) 121 (18.2%) | 0.0066 0.58               <0.0001 0.37 | |
| Depressive symptoms | 7.67 | 4.93 | <0.0001 | 9.73 | 7.10 | <0.0001 | |
| Anxiety symptoms | 18.34 | 15.79 | <0.0001 | 20.83 | 18.49 | <0.0001 | |
| Wellbeing | 4.28 | 5.06 | <0.0001 | 3.80 | 4.36 | <0.0001 | |
| Adaptive coping  Psychological care   *Yes  Waiting list* | 3.85  18 (21.7%) 2 (2.4%) | 4.31  47 (12.0%) 8 (2.04%) | 0.0042 0.084 | 3.79  57 (38.0%) 11 (7.3%) | 4.09  115 (17.3%) 29 (4.4%) | 0.0079 0.00083 | |
| ESM | 39.64 | 51.51 | 0.0059 | 37.47 | 36.10 | 0.57 | |
| Challenge | 15.32 | 17.49 | 0.059 | 12.04 | 11.18 | 0.05 | |
